# Big Tau: Structure, Evolutionary Divergence, and Emerging Roles in Cytoskeletal Dynamics and Tauopathies

**DOI:** 10.3390/cells15030241

**Published:** 2026-01-27

**Authors:** Itzhak Fischer, Peter W. Baas

**Affiliations:** Department of Neurobiology and Anatomy, Drexel University College of Medicine, 2900 Queen Lane, Philadelphia, PA 19129, USA; pwb22@drexel.edu

**Keywords:** tau, microtubule-associated protein, neurons, exon 4a, hydrophobicity, protein aggregation, neurodegeneration, evolutionary conservation

## Abstract

**Highlights:**

**What are the main findings?**

- Inclusion of the large exon 4a insert generates Big tau, a high-molecular-weight isoform of tau that predominates in the peripheral nervous system and in select regions of the central nervous system.
- Exon 4a encodes about 250 amino acids that form a highly acidic, intrinsically disordered domain whose length is evolutionarily conserved despite marked divergence in the primary sequence.

**What are the implications of the main findings?**

- Modeling suggests that the expansion of the projection domain in Big tau sterically and electrostatically shields β-sheet-forming regions implicated in pathological fibril assembly.
- Elucidating how this structural adaptation affects axons and suppresses tau aggregation provides insight into selective neuronal vulnerability and may inform strategies to engineer aggregation-resistant tau variants.

**Abstract:**

Tau proteins are microtubule-associated proteins that regulate axonal structure, dynamics, and transport, and their dysregulation underlies several neurodegenerative diseases. The *MAPT* gene produces multiple tau isoforms through alternative splicing, including the high-molecular-weight isoform known as Big tau, which contains an insert of the large 4a exon of approximately 250 amino acids. Big tau is predominantly expressed in neurons of the peripheral nervous system (PNS), cranial motor nuclei, and select neurons of the central nervous system (CNS) such as the cerebellum and brainstem. Developmental expression studies indicate a switch from low-molecular-weight isoforms of tau to Big tau during axonal maturation, suggesting that Big tau optimizes cytoskeletal dynamics to accommodate long axonal projections. Comparative sequence and biophysical analyses show that the exon-4a insert is highly acidic, intrinsically disordered, and evolutionarily conserved in its length but not its primary sequence, implying a structural role. Emerging modeling and in vitro assays suggest that the extended projection domain provided by the exon-4a insert spatially and electrostatically shields the aggregation-prone PHF6 and PHF6* motifs in tau’s microtubule-binding domain, thereby reducing β-sheet driven aggregation. This mechanism may explain why tauopathies that involve aggregation of tau have little effect on the PNS and specific regions of the CNS such as the cerebellum, where Big tau predominates. Transcriptomic and proteomic data further suggest that alternative Big tau variants, including 4a-L, are expressed in certain cancerous tissues, indicating broader roles in cytoskeletal remodeling beyond neurons. Despite its putative anti-aggregation properties, the physiological regulation, interaction partners, and in vivo mechanisms of Big tau remain poorly defined. This review summarizes what is known about Big tau and what is missing toward a better understanding of how expansion via inclusion of exon 4a modifies tau’s structural and functional properties. Our purpose is to inspire future studies that could lead to novel therapeutic strategies to mitigate tau aggregation in neurodegenerative diseases.

## 1. General Background

Tau is encoded by the MAPT gene, which is composed of 14 exons (exons 1–13, with 4a being an alternative exon expressed in Big tau) and characterized originally as a microtubule-associated protein (MAP) with a wide range of orthologs in vertebrates and invertebrates [1,2]. Tau proteins have been shown to modulate the dynamic properties of microtubules (MTs) and axonal transport, shape the structure of neurons, and play a central role in neurodegeneration [3,4,5]. The structure and molecular properties of canonical low-molecular-weight (LMW) tau are determined by 13 exons that generate six isoforms (of apparent molecular weights of 45–60 kDa) by alternative splicing of exons 2, 3 at the N-terminal and exon 10 in the MT-binding domain (MTBD). Domains of tau, which include the N-terminal projection region, the proline-rich region, the MTBD, and the C-terminal region, have different biophysical and functional properties [6,7,8].

**Figure 1:**
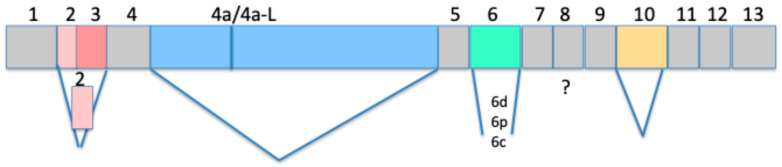
Exon structure of Big tau. The schematic shows the exon structure of Big tau, which includes the canonical representation of exons 2/3 (N-terminal), 4a (Big tau), 6, and 10 (MTBD), which are colored. The diagram also shows the known alternative splicing at the N-terminal with either exons 2 and 3, exon 2 alone or none, exon 4a (right blue section) or its longer form of 4a-L (complete blue section) defining Big tau, exon 6 with 3 splice sites (6c, 6p, and 6d) labeled, exon 8, whose expression is poorly understood, and exon 10, which is one of the MTBDs defining the 3R/4R isoforms.

A larger isoform termed Big tau [9,10] includes an additional exon, termed 4a, to form a protein with an apparent molecular weight of 110 kDa (Figure 1). Early studies of Big tau revealed its unique distribution in neurons of the peripheral nervous system (PNS) and select neurons of the central nervous system (CNS) [11] and provided some insight into the physiological properties of Big-tau-expressing neurons [12]. More recent studies demonstrated that neurons expressing Big tau originally expressed LMW tau isoforms and then transitioned developmentally to express Big tau early in postnatal life [12].

Here, we review the expression of Big tau in neural and non-neural tissue, provide an evolutionary perspective on the structural changes in tau provided by the inclusion of exon 4a, and discuss the possible functional consequences. We offer an integrated view of sequence conservation, structural modeling, and biochemical analyses to elucidate how Big tau achieves aggregation resistance and contributes to neuronal resilience. We also propose a working structure/function model and discuss the necessary experimental steps to bridge the missing knowledge gaps (summarized in Supplementary Materials Table S1). Understanding these issues may illuminate the evolutionary diversification of tau and identify protective features relevant to neurodegenerative disease.

## 2. Expression of Big Tau in PNS and CNS

### 2.1. Sensory Root Ganglion (DRG) Sensory Neurons

Most sensory ganglia such as rat DRG express Big tau [11,13]. During development at early embryonic stages, they initially express LMW tau, but later Big tau becomes the primary isoform in DRGs and dorsal roots. The distribution of Big tau appears to be selective to adult small and medium-sized neurons (IB4 and CGRP-positive) but not large neurons (parvalbumin-positive).

### 2.2. Autonomic Neurons (Superior Cervical Ganglion, SCG)

SCG neurons express in early embryonic stages the LMW isoforms of tau but undergo a transition to the Big tau isoform, which is completed by 4–5 weeks postnatally. Regeneration experiments with SCG explants suggest that the expression of Big tau does not correlate with regeneration but rather with the structure of mature SCG neurons [14], consistent with sciatic nerve regeneration experiments where Big tau decreases following axotomy, and that the tau isoform pattern remains adultlike [13].

### 2.3. Cranial Nerves

In the adult CNS, most brainstem neurons that project to the PNS express Big tau, including motor nuclei of the oculomotor, trochlear, trigeminal, abducens, facial, glossopharyngeal, vagus, and hypoglossal [11]. In the spinal cord, the expression of Big tau is detected in dorsal roots and in spinal motor neurons including ventral roots. Again, during development, the LMW forms of tau are replaced by Big tau in early postnatal time. These data have potential applications for Big tau as a biomarker for motor neurons in anatomical analysis and for understanding the pathology of motor neuron degeneration such as Amyotrophic Lateral Sclerosis (ALS) [15].

### 2.4. Visual System Retinal Ganglion Cells (RGCs)

The presence of Big tau in rat RGCs was previously reported [11,16] in studies showing labeling of RGCs and the optic nerve at 90 kDa (e.g., middle MW tau isoform). Axonal transport studies [12,17] showed that adult optic nerve contained both LMW tau and the middle MW form of Big with transport rates distinct from that of tubulin and other MAPs. Our studies [18] have shown that RGCs express Big tau that includes not only exon 4a but also exon 6 and exon 10. However, they lack exons 2 and 3 at the N-terminal, which may account for the lower MW of 90 kDa. Adult RGC and the optic nerve express high levels of LMW tau and lower levels of the 90 kDa form of Big tau, which appears to be present in all RGCs and their axons.

### 2.5. Cerebellum

The expression of Big tau in the cerebellum has been reported in several studies [11,16,19] with staining restricted to the Purkinje neuron somata and proximal dendrites. Western blot analyses have revealed that Alzheimer’s disease (AD) patients show a higher expression level of Big tau in the cerebellum, the brain region typically spared from tau pathology, suggesting that some types of neurons may upregulate Big tau levels as a protective reaction to disease [19].

## 3. Expression of Big Tau in Non-Neural Tissue

Skeletal Muscle: Multiple studies (RT-PCR, transcript cloning, some protein blots) report exon-4a-containing MAPT transcripts and Big-tau-sized proteins bands [20,21,22].

Heart: A recent study indicates Big tau expression in the heart, with Big tau species becoming toxic in the diseased myocardium [23]. These studies identify Big tau as the predominant isoform in the heart, show that it becomes hyperphosphorylated and aggregates in heart failure (HF) and AD, and proposes that the mechanism is disrupted MT tyrosination leading to myocardial stiffness/diastolic dysfunction. The authors also report that monoclonal anti-tau antibody therapy improved myocardial function and cleared the aggregates. These results provide a line of evidence that Big tau is not completely unable to become toxic and contribute to disease, at least in non-nervous tissues.

Lung: A recent study [24] reported that lung endothelial cells contain both LMW tau isoforms and Big tau with four MTBD. Infection of the endothelial cells with a virulent Pseudomonas aeruginosa promoted the release of cytotoxic tau, leading to tau aggregation in reporter HEK293 cells.

## 4. Control of Alternative Splicing

There have been excellent reviews on the control of alternative splicing of the six canonical isoforms of tau with respect to exons 2, 3, and 10 [22,25,26,27]. Here, we will focus on the splicing of exon 4a and 6 associated with the expression of Big tau. Exon 4a is present primarily in the PNS and select regions of the CNS, and exon 6 is found primarily in the PNS and spinal cord. In both cases, the size of the resulting protein is determined by the choice of 3′ splice site. There are two 3′ splice sites for the 4a exon, generating exon 4a and 4a-L (discussed later). The 4a exon is excluded by default splicing patterns requiring an activator for its inclusion, but the precise splicing mechanism remains unclear [28]. Exon 4a is often included together with exon 6, but it has also been found as an isoform without exon 6 and also without exons 2 and 3, usually with exon 10 [12].

Exon 6 has three splice sites generating variants of 6c, 6p, and 6d (also discussed later). Studies by Andreadis’s group have demonstrated that all combinations of exons 6c, 6p, and 6d with the other alternative exons are possible [29]. The inclusion of exons 6p and 6d includes a frameshift resulting in the translation of a truncated protein lacking MT-binding sites because of a change in the reading frame that introduces a stop codon.

## 5. Evolutionary Analysis of Big Tau

The 4a exon, which defines Big tau, appears to have arisen early in vertebrate evolution as a large insert that dramatically increased the size of the tau protein. Despite its relatively consistent length of ~250 amino acids (and in some species a larger 4a-L variant of ~355 amino acids), its primary sequence conservation (at the protein level) is remarkably low. Across vertebrates, the 4a exon shows only 17–25% identity in non-mammals including birds, reptiles, amphibians, and fish; intermediate conservation in non-primate mammals (50–60%); and the highest conservation among primates at 90–100% [30]. This pattern suggests that this exon may have retained biophysical properties (size, net charge, hydrophilicity) rather than strict sequence homology. We previously proposed that exon 4a may have arisen through exonization, the process by which novel sequences were incorporated into *MAPT* via alternative splicing to meet the structural and functional demands of increasingly complex nervous systems. Thus, the evolution of a significantly larger tau isoform appears to have occurred independently multiple times, highlighting convergent selective pressure across vertebrates for an elongated projection domain that may provide both novel physiological functions and protection against aggregation.

To extend these findings, we systematically analyzed conservation of all 13 canonical tau exons (plus 4a) across six representative vertebrate species: a mammal, a bird, an amphibian, and three groups of fishes (Supplementary Materials Table S2). As expected, exon 4a showed the lowest sequence conservation, particularly in fish where identity was near background levels, though its size remained within ~250 amino acids. By contrast, the N-terminal exons 1–4 (with exons 2 and 3 alternatively spliced) and the projection domain (exons 5–8) displayed moderate conservation, reaching ~80% identity in mice and 15–20% in fish. As expected, the MT-binding domain (MTBD, exons 9–12 and exon 14) was the most conserved region, with sequence identities in the high 90%s in mammals, ~80% in amphibians and bony fishes, and up to ~50% even in the primitive jawless lamprey. As expected, primates had an almost identical sequence identity to humans and even the 4a exons showed >95% sequence identity. These repeats also retain strong homology with invertebrate tau-like proteins, including *Drosophila*, whose tau-like protein preserves the critical PHF6 (VQIVYK) and PHF6* (VQIINK) motifs. Interestingly, the C-terminal exon 13 was also highly conserved across vertebrates, suggesting an under-recognized functional role beyond the MTBD. In the jawless vertebrate lamprey, exon mapping was often ambiguous due to deep evolutionary divergence and differences in exon–intron organization of MAPT, consistent with the long evolutionary distance separating Cyclostomes from jawed vertebrates. Nonetheless, detectable homology within the MTBD suggests that the core tau–MT interaction has been preserved since the earliest vertebrates.

## 6. Big Tau Expression in Cancer

Tau is expressed in some tumors and has been studied for its role in resistance to MT-targeting agents (e.g., paclitaxel/taxol), since tau competes with these drugs on the MT lattice [31]. Big tau has been reported in certain cancer transcriptomes, especially in contexts where aberrant splicing of MAPT occurs. Big tau with the 4a-L (the longer variant of exon 4a) has been observed in RNA-seq datasets from some tumor types, and specifically in cells derived from prostate cancer [32]. It is possible that the long projection domain of 4a-L reduces tau aggregation risk but also lowers MT binding strength, which could help cancer cells maintain dynamic, remodelable MTs needed for rapid proliferation and migration. The 4a-L isoform may confer a protective shield against aggregation stress in highly metabolic tumor environments and modulate sensitivity to MT poisons (taxanes, vinca alkaloids), a key therapeutic resistance mechanism. It is not clear, however, whether 4a-L expression is specific to certain cancer types (gliomas vs. epithelial cancers) or can serve as a marker of progression, aggressiveness, or drug resistance. Does it play a causal role, or is it merely a byproduct of splicing dysregulation common in tumors? If the latter, then therapeutics aimed at reducing, degrading, or otherwise disabling Big tau might be useful in combination with other therapies for treatment of certain cancers. Ongoing projects to harness big data in cancer research [33] may reveal correlations of different tau isoforms with types of cancer and possible therapeutic pathways.

## 7. Big Tau as Biomarker

Based on the properties of Big tau of selective expression in spinal motor neurons, but not in upper motor neurons or other spinal neuronal populations, we proposed that it could be a specific biomarker for spinal motor neuron pathology [15]. This expression pattern may be particularly valuable for tracking disease prognosis and progression in conditions such as ALS and related disorders, to identify when degeneration advances to lower motor neurons. These include other neurological disorders such as Spinal Muscular Atrophy (SMA) and Progressive Muscular Atrophy (PMA), as well as infectious diseases such as Poliomyelitis (Polio) and West Nile virus. Big tau could thus serve as a more specific biomarker than neurofilament or LMW tau proteins or can be used in combination with other biomarkers such as neurofilaments [34] to enhance the specificity and sensitivity. Specific Big tau antibodies are needed to validate the role of Big tau as a biomarker, and we encourage clinicians to develop ELISA protocols to test this proposition.

## 8. New Data on 4a Structure

In recent review articles [12,35,36], we suggested that the increased length of the projection domain of Big tau may affect the conformational changes with a lower propensity to polymerize into fibrils compared with LMW variants of tau and may be less able to be released from the neuron and propagate from neuron to neuron. This could explain why tauopathies that prominently affect the brain have little or no comparable impact on the PNS, with only relatively rare reports of tau tangles in peripheral neurons and in CNS regions such as the cerebellum. Our evolutionary studies indicated that despite low sequence homology the length of exon 4a that defines Big tau is conserved and remains at about 250 amino acids across vertebrate species. However, we lacked data on the structural properties of the 4a exon that contribute to the reduced aggregation propensity of Big tau.

Recently, we analyzed the charge distribution, hydrophobicity, and aggregation propensity of the human sequences of LMW tau, Big tau, and the stretch of amino acids encoded by exon 4a [37]. We found that the composition of the exon 4a encoded stretch is intrinsically disordered, enriched for acidic amino acids with a net negative charge, together with a hydrophilic composition, as well as a low β-sheet content. In contrast, the LMW tau has a lower distribution of acidic amino acids (ratio of negative to positive aa of 1.1 vs. 1.3 for Big tau), is more hydrophobic (hydropathy value of −0.893 vs. −0.925 for Big tau), and contains extended aggregation-prone motifs within the MT-binding domain including high β-sheet content (17.33% vs. 4.78 for Big tau) calculated using AggreProt and Aggrescan. The inclusion of exon 4a in Big tau shifts the global hydrophobicity to intermediate values and reduces predicted β-sheet content, suggesting decreased aggregation potential. We propose that it creates steric hindrance and a protective umbrella that reduces the chance that the MT-binding repeat domains (which contain the PHF motifs) come into proximity and self-assemble. Evolutionary analyses across mammals, birds, and amphibians confirms the minimal sequence identity and conserved size of exon 4a but also the preservation of net negative charge and hydrophobicity, indicating retention of the biophysical properties that reduce aggregation propensity.

Previous studies have shown that the core aggregation structures of tau are the PHF6* (VQIINK) and PHF6 (VQIVYK) sequences, which are short hexapeptide motifs in the repeat domain of MT binding encoded by exons 10 and 11 [38,39]. Both are hydrophobic and rich in β-sheet-forming residues, and when these sequences are exposed (i.e., tau is unbound to MTs), they readily form β-strand structures that can stack into cross-β fibrils seen in tauopathies. It is also known that the N-terminal sequences which are negatively charged and highly hydrophilic have the potential to shield the repeats, reducing aggregation. Indeed, biophysical studies [40,41] suggest tau adopts a “paperclip” or folded conformation in solution: the N-terminal region folds back over the repeat domain. The C-terminal tail folds forward to contact repeat domains [42]. This folding partially buries the PHF6 and PHF6 motifs*, reducing their exposure to solvent and other tau molecules, and as a result, aggregation nucleation is slowed under normal conditions. However, disease-associated modifications (hyperphosphorylation, truncation) expose PHF6 and PHF6*, making them aggregation-prone.

Building on the anti-aggregation properties of the N-terminal, we proposed that addition of the 250 amino acids to the projection domain adjacent to the N-terminal keeps the repeat domains of neighboring tau molecules further apart, reducing the likelihood of β-sheet stacking acting as a shield to prevent tau–tau interactions that drive aggregation and PHF formation. The combined effects may provide protection even under pathological conditions by increasing the spacing between the MT-binding repeats and neighboring tau molecules, reducing the likelihood of β-sheet stacking and providing a hydrophilic shield. It may also reduce accessibility to phosphorylation sites associated with pathological hyperphosphorylation.

## 9. Why Are the Aggregation Rich Domains of the Repeats Conserved?

The MTBD repeats are essential for MT binding. They mediate tight MT interactions but also self-assembly. Evolution has conserved these repeats to maintain MT function, despite the aggregation risk, which usually rises with age, a time that is less under evolutionary pressure. Specifically, tauopathies like AD or ALS typically develop after reproductive age while evolution “cares” about fitness until reproduction; diseases that appear later exert weak selective pressure. Indeed, the aggregation-prone sequence is largely harmless during development and reproductive life, so natural selection does not eliminate it. Furthermore, the PHF6*/PHF6 domains contain alternating hydrophobic and polar residues that allow reversible MT binding under physiological conditions. Once the balance is disturbed (phosphorylation, truncation, oxidative stress), hydrophobic patches dominate, promoting nucleation of fibrils. Another factor is exon 10 (R2) that amplifies aggregation (e.g., altered 3R:4R tau ratio in tauopathies).

The β-sheet-prone residues that mediate aggregation are the same residues that allow tight MT binding. Any mutation to reduce aggregation would likely weaken tau–MT interactions, which is highly deleterious. Evolution cannot “solve” aggregation risk without compromising tau function, which is a common functional constraint. There are examples of such trade-offs, such as the amyloid-beta precursor protein, and some transcription factors have aggregation-prone regions.

## 10. Why Is Big Tau Not the Dominant Form in CNS?

The absence of Big tau as the dominant form in the CNS likely reflects a convergence of structural, functional, metabolic, and evolutionary pressures. One major factor lies in axonal architecture and MT spacing. Big tau is much larger at 110 kDa than the LMW isoforms at 45–60 kDa because of the large exon 4a insert, which adds roughly 250 amino acids to the projection domain. This extension can increase the spacing between adjacent MTs, a property that is advantageous in long, thick-caliber axons such as those of peripheral nerves and spinal motor neurons, where wide MT separation supports robust and sustained axonal transport. In contrast, in the shorter, fine-caliber axons and dendrites of the CNS, this wider spacing would disrupt normal MT packing and interfere with the precise organization required at synapses.

Functional specialization further explains the preference for LMW tau in the CNS. LMW isoforms are highly dynamic, capable of rapidly binding and releasing from MTs. This plasticity underpins synaptic remodeling, activity-dependent cytoskeletal reorganization, and processes essential for learning and memory. Big tau, by contrast, affords less plasticity, and this comes at the expense of adaptability. While less plasticity is suited to maintaining long-distance axons, it is less compatible with the ever-changing cytoskeletal requirements of cortical and hippocampal neurons. Another important constraint is the metabolic and spatial cost of producing and trafficking such a large protein. Synthesis and transport of the much larger isoform are energetically demanding, and in neurons with short axons but high synaptic density—such as cortical interneurons—the energetic cost outweighs any stabilizing advantage. From an evolutionary perspective, neurons have selected isoforms that optimize the balance between less plasticity and more adaptability for their specific morphology and functional role.

**Figure 2:**
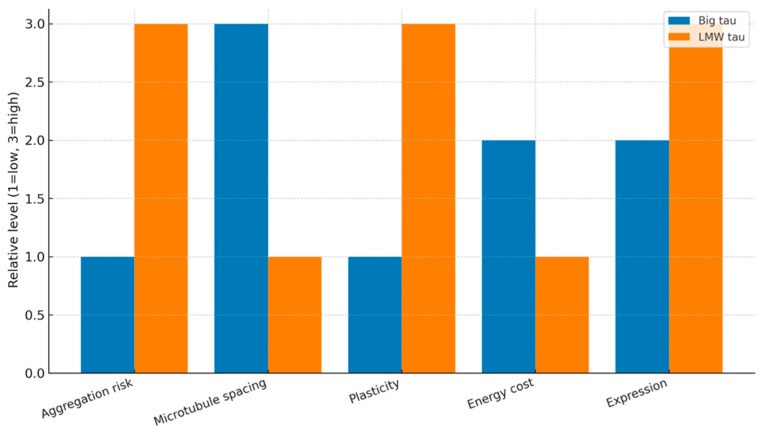
Big tau vs. LMW tau—evolutionary trade-offs. A summary figure that approximates the properties of Big tau and LMW tau in an evolutionary perspective. It illustrates the trade-off and adaptation of the proteins into different physiological functions affecting clinical consequences. Note that these are qualitative estimates of low and high derived from our Section 12, Section 13 and Section 14.

The contrast between Big tau and LMW tau also illustrates a broader evolutionary trade-off between protection and plasticity. Big tau, with its acidic, hydrophilic, and intrinsically disordered 4a insert, resists aggregation, offering a degree of protection against tauopathy-like misfolding. However, this protection comes at the expense of the fine-tuned cytoskeletal regulation needed in the CNS. LMW tau, however, is more vulnerable to aggregation, particularly with aging or under disease conditions, but it enables rapid and dynamic MT remodeling critical for cognitive flexibility. Evolution appears to have prioritized plasticity over aggregation resistance, particularly because most tauopathies manifest late in life, after the reproductive period, when evolutionary pressure is weak. Studies are underway by our team that will provide experimental evidence for these ideas (see later). These considerations suggest that the limited distribution of Big tau is not coincidental but rather reflects incompatibility with the specialized demands of CNS neurons (summarized in Figure 2).

## 11. Analysis of Exon 4a vs. 4a-L

**Figure 3:**
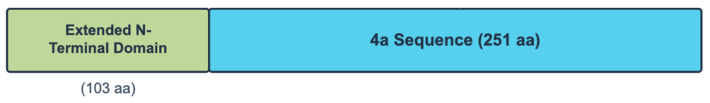
Exon 4a vs. 4a-L. A diagram showing the structural relationship between the 4a and 4a-L exons in the composition of human tau and the difference in size of 251 amino acids for 4a vs. 354 for 4a-L.

Big tau has an alternative form of the 4a exon termed 4a-L (Figure 3), which includes an additional N-terminal segment before the canonical 4a sequence and has so far been found to be expressed only in prostate cancer cells [43].

As recently described [30], the analysis of the MT-associated protein tau (MAPT) gene using Ensembl was carried out by examining the MAPT GeneTree that contains four speciation nodes with protein sequences annotated for predicted exons that include birds, turtles, alligators, lizards, mammals, and amphibians. Big tau with a canonical 4a exon is widely found across vertebrates, while the longer 4a-L variant in present only in several species (notably primates and some other mammals) and was shown to be expressed only in cancer cell lines. The sequence of the 4a-L exon, like that of the canonical 4a, is highly diverged regarding sequence homology but retains a consistent size of about 350 amino acids. The following section presents the analysis of the biophysical properties of the 4a-L exon in comparison to the canonical 4a.

### 11.1. Charge Distribution—Result: Net Negative Charge 4a (1.303) > 4a-L (1.196)

Canonical 4a has a strongly acidic bias with an overall negative net charge. This charge distribution promotes electrostatic repulsion and helps keep the repeat domains of tau physically separated, reducing aggregation tendency [44]. The 4a-L variant retains the acidic/polar character, but the extension introduces more mixed regions with patches of neutral or basic residues. This results in a less uniform charge distribution with a potential to form local charge-neutral clusters, which could allow occasional self-association. Thus, 4a-L may be less efficient than 4a-short in maintaining spacing between PHF repeat domains.

### 11.2. Hydrophobicity = 4a (−0.926) Is More Hydrophilic than 4a-L (−0.879)

The 4a exon is overall extremely hydrophilic and may function like an intrinsically disordered “spacer” domain that avoids self-association [44]. 4a-L is also hydrophilic overall on average, but the extended N-terminal segment adds slightly more hydrophobic patches, which could form weak intermolecular interactions. This may explain why 4a-L shows up more in dysregulated cancer splicing—a more aggregation-prone context.

### 11.3. β-Sheet/Aggregation Propensity

This value represents the fraction of residues in the protein that structurally favor the formation of a β-sheet (a structure crucial for aggregation).

The 4a exon-derived protein has very low predicted β-sheet content with sequence composition that acts as a disordered, protective spacer. 4a-L is still largely disordered, but computational predictions suggest slightly increased β-strand propensity compared to 4a. This could modestly increase aggregation risk. This potentially renders it less protective than 4a, possibly explaining its absence from long peripheral axons and sporadic appearance in CNS/cancers.

However, while the amino acid composition is slightly more aggregation-prone, the actual aggregation tendency may be reduced by the steric and dynamic shielding of the extended N-terminal domain, a property not captured by simple fractional counts.

## 12. Conceptual Model of Big Tau’s Protective Mechanism

### 12.1. Structural Overview

**Figure 4:**
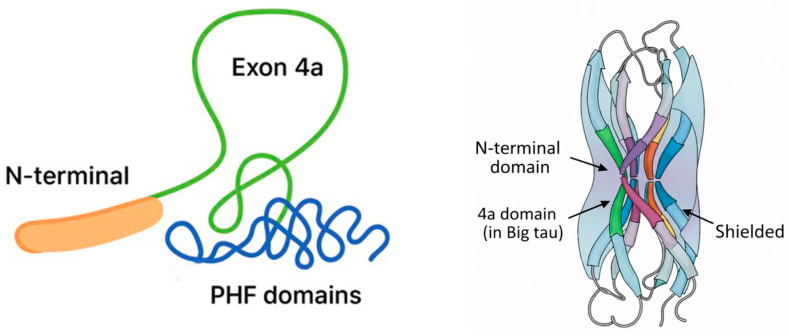
Diagrams of the shielding effects of Big tau. The simplified diagrams illustrate how the Big tau structure can shield the aggregation regions by having a large projection domain that includes the N-terminal and the 4a exon (**left panel**) which are hydrophilic and carry negative charges providing a steric and electrostatic shield (**right panel**). The 3D structure of Big tau predicted by AlphaFold is shown in [44].

Structural analysis of the canonical LMW tau associated with neurodegeneration has shown that distinct conformers of filamentous tau define different tauopathies [45] and are affected by posttranslational modifications [46]. Big tau at 110 kDa differs from canonical LMW tau by inclusion of the large exon 4a insert of about 250 amino acids. This insert expands the N-terminal projection domain, extending tau’s overall length and separating the MTBD from neighboring tau molecules (Figure 4).

### 12.2. Shielding Mechanism

The 4a encoded protein is intrinsically disordered, acidic, and hydrophilic, forming a negatively charged domain, which together with the N-terminal forms a flexible shield around the MTBD region. This region provides electrostatic and steric shield, reducing the likelihood that the aggregation-prone PHF6 (VQIVYK) and PHF6 (VQIINK)* motifs in the MTBD will interact with other tau molecules. The enlarged projection domain likely increases intermolecular spacing between adjacent tau-bound MTs, which may also lower aggregation potential, as well as reducing MT-binding affinity due to shielding of the MT-binding sites (depicted schematically in Figure 4).

### 12.3. Biophysical Properties

Charge: Enrichment of glutamate and aspartate residues in exon 4a results in a strong net negative charge, repelling other tau molecules. Hydrophobicity: Inclusion of exon 4a decreases overall hydrophobicity and β-sheet content, which are critical drivers of tau–tau aggregation. Conformation: The large projection domain supports a more extended configuration, limiting exposure of aggregation motifs.

### 12.4. Functional Implications

Reduced aggregation propensity: Big tau resists formation of aggregates under conditions that promote LMW tau aggregation. Neuroprotective role: the extended domain likely minimizes pathological aggregation of tau, thus minimizing tauopathies and neuronal degeneration. Physiological trade-off: while protective, Big tau’s rigid structure and large spacing may reduce MT adaptability appropriate to long, less plastic axons of peripheral neurons rather than highly plastic CNS circuits. Reduced MT-binding affinity of Big tau compared to LMW tau may also contribute to this effect by enabling greater binding to MT of bona fide MT-stabilizing proteins such as MAP6 [47,48].

### 12.5. Evolutionary Insights

The appearance of the 4a exon, whose sequence homology is low but whose length is consistent, early in vertebrate evolution suggests repeated, independent exonization events providing similar protective and structural roles across vertebrate species. Despite low sequence conservation, the size, charge, and disordered nature of exon 4a are evolutionarily preserved—indicating strong selective pressure for its biophysical function rather than specific amino acid sequence.

## 13. What Is Still Missing with Respect to Experimental Evidence

What has already been shown experimentally is the analysis of Big tau expression in PNS and select regions of CNS (Section 2), the computational analysis of the evolutionary structure of the 4a in vertebrates (Section 3), and its biophysical properties (Section 4).

There have also been studies that analyzed Big tau expression following nerve regeneration [13] and a preprint on the consequences of transgenic animals expressing Big tau [19]. Ongoing work by our team involves cultures of rat hippocampal neurons transfected to express low-molecular-weight (LMW) human tau bearing two pathological mutations that result in formation of toxic tau aggregates that cause degeneration of the neurons. When the mutant tau construct included exon 4a (thus making it Big tau), we are finding significantly less tau aggregation and no indication of toxicity, suggesting that neurons that express Big tau are less prone to tau pathology (manuscript in preparation).

### 13.1. Tau Aggregation Assays (In Vitro)

Studies using recombinant tau fragments show that longer N-terminal and central inserts reduce aggregation propensity [49]. What is left is to perform these experiments with neuronal cell culture and recombinant Big tau using various sizes of the 4a exon to determine the critical size that provides the optimal protection (see above, ongoing work by our team).

### 13.2. Lack of In Vivo Functional Models

There is a need to develop transgenic models of knock-in mouse expressing Big tau in the CNS to test protection against aggregation and tauopathy. Such a model could also examine possible effects on altered dendritic and synaptic morphology, potentially impairing learning-related plasticity. This trade-off is probably why evolution restricted Big tau to PNS and only certain CNS neurons (see above preprint data).

### 13.3. Molecular Mechanism of Aggregation Resistance

Our data suggest exon 4a reduces the aggregation propensity of Big ta and may sterically shield PHF6/PHF6* motifs, but direct structural data (immuno-EM of Big tau filaments) are lacking. Experimental data may also reveal alternative mechanisms in which Big tau alters phosphorylation or turnover rather than direct steric shielding.

### 13.4. Expression of Exon 6

The expression of exon 6 is uncommon, complex, and controversial, and its function remains unknown [26,28,50]. It is mainly present in peripheral neurons that express Big tau. There have been studies that identify low levels of the canonical isoforms of tau isoforms expressing exon 6 in the brain, but they are particularly prominent in Big tau expressed in skeletal muscle and the spinal cord. The splicing behavior of exon 6 is complex and may include two alternative 3′ splice variants, (6p/6d), which cause a frameshift that generates truncated proteins [29]. The functions of these shorter tau isoforms remain unknown, but they are less prone to form aggregates and inhibit the aggregation of full-length tau protein as they lack the MBTD, which is needed for aggregation [51]. Interestingly, these isoforms can be found at the highest levels in the spinal cord and cerebellum regions where neurofibrillary tangles are absent, even in patients with neurodegenerative disorders. Regarding known mutations, pathogenic/FTD-linked MAPT variants are overwhelmingly found in the repeat region of exon-10 and flanking splice sites [52], but there are no clinical pathogenic hits in exon 4a.

### 13.5. Functional Consequences for Axonal Biology

Big tau is expressed in PNS axons possibly to facilitate long-distance transport and spacing. However, there is no experimental evidence that Big tau actively promotes transport efficiency or that the vulnerable PNS axons which are under metabolic stress prioritize less plasticity and protection against aggregation while not requiring CNS-level plasticity. Immuno-EM analysis of axons with Big tau may reveal increased MT spacing (relative to axons with LMW tau) due to its large projection domain, creating a wider axonal cytoskeleton lattice. This spacing may reduce crowding and allow more efficient vesicle and organelle transport, particularly in long PNS axons.

### 13.6. Role in Human Disease

Big tau expression has not been reported in AD or Frontotemporal Dementia (FTD) pathology. It is however unknown whether loss of Big tau expression in PNS will promote peripheral neuropathies or whether knowledge of Big tau can be harnessed therapeutically to reduce CNS tau aggregation. Big tau expression in CNS could theoretically prevent tau aggregation, but it may impair synaptic plasticity if expressed in cortical or hippocampal neurons. Therapeutically, it is also possible that targeted expression in vulnerable long projection CNS neurons might be protective without global CNS disruption. A more focused therapeutic strategy can be devised from the properties of the 4a exon that defines Big tau as a module with a negative net charge of acidic amino acids, with an overall hydrophilic composition. Similar modifications could emulate Big tau’s resistance to misfolding without disrupting MT binding.

## 14. Conclusions

The evolutionary persistence of Big tau highlights a critical biological trade-off between neuroplasticity and tau’s propensity for aggregation and disease. While the canonical LMW tau isoforms enable the rapid cytoskeletal remodeling required for complex CNS functions, they come at the cost of heightened susceptibility to aggregation and neurodegeneration. In contrast, the inclusion of exon 4a in Big tau creates a disordered, electrostatic shield that protects long projecting neurons from the pathological aggregation of tau central to the pathology of AD and other tauopathies. Future research must now move to rigorous in vivo modeling and high-resolution structural assays to determine the role of Big tau in the nervous system so that knowledge can be applied to treatment strategies of neurons prone to those diseases.

## Data Availability

The original contributions presented in this study are included in the article/Supplementary Material. Further inquiries can be directed to the corresponding author.

## Supplementary Materials

The following supporting information can be downloaded at https://www.mdpi.com/article/10.3390/cells15030241/s1, Table S1: Summary. This is a graphical abstract of the review with a summary of the differences between Big tau and LMW tau. Table S2: Exon-by-exon conservation table (human MAPT exons 1 → 13). The table, which shows the approximate sequence identity of different vertebrate species relative to human tau exon by exon, illustrates the very low conservation of exon 4a and the high conservation of the MTBD. The approximate ranges are simplified in presentation for the different vertebrate orders clustered into five general domains.

## References

[1] Sundermann F., Fernandez M.P., Morgan R.O. (2016). An evolutionary roadmap to the microtubule-associated protein MAP Tau. BMC Genom..

[2] Dehmelt L., Halpain S. (2005). The MAP2/Tau family of microtubule-associated proteins. Genome Biol..

[3] Tapia-Rojas C., Cabezas-Opazo F., Deaton C.A., Vergara E.H., Johnson G.V.W., Quintanilla R.A. (2019). It’s all about tau. Prog. Neurobiol..

[4] Morris M., Maeda S., Vossel K., Mucke L. (2011). The many faces of tau. Neuron.

[5] Wegmann S., Biernat J., Mandelkow E. (2021). A current view on Tau protein phosphorylation in Alzheimer’s disease. Curr. Opin. Neurobiol..

[6] Brandt R., Trushina N.I., Bakota L. (2020). Much More Than a Cytoskeletal Protein: Physiological and Pathological Functions of the Non-microtubule Binding Region of Tau. Front. Neurol..

[7] Wang Y., Mandelkow E. (2016). Tau in physiology and pathology. Nat. Rev. Neurosci..

[8] Alonso A.D.C., El Idrissi A., Candia R., Morozova V., Kleiman F.E. (2024). Tau: More than a microtubule-binding protein in neurons. Cytoskeleton.

[9] Goedert M., Spillantini M.G., Crowther R.A. (1992). Cloning of a big tau microtubule-associated protein characteristic of the peripheral nervous system. Proc. Natl. Acad. Sci. USA.

[10] Georgieff I.S., Liem R.K., Couchie D., Mavilia C., Nunez J., Shelanski M.L. (1993). Expression of high molecular weight tau in the central and peripheral nervous systems. J. Cell Sci..

[11] Boyne L.J., Tessler A., Murray M., Fischer I. (1995). Distribution of Big tau in the central nervous system of the adult and developing rat. J. Comp. Neurol..

[12] Fischer I. (2024). Big tau: What, how, where and why. Cytoskeleton.

[13] Oblinger M.M., Argasinski A., Wong J., Kosik K.S. (1991). Tau gene expression in rat sensory neurons during development and regeneration. J. Neurosci..

[14] Jin Y., Connors T., Bouyer J., Fischer I. (2023). Regulation of Tau Expression in Superior Cervical Ganglion (SCG) Neurons In Vivo and In Vitro. Cells.

[15] Fischer I. (2025). Considering Big tau as a novel and specific biomarker for spinal motor neuron pathology. Neurobiol. Dis..

[16] Taleghany N., Oblinger M.M. (1992). Regional distribution and biochemical characteristics of high molecular weight tau in the nervous system. J. Neurosci. Res..

[17] Mercken M., Fischer I., Kosik K.S., Nixon R.A. (1995). Three distinct axonal transport rates for tau, tubulin, and other microtubule-associated proteins: Evidence for dynamic interactions of tau with microtubules in vivo. J. Neurosci..

[18] Fischer I., Connors T., Bouyer J., Jin Y. (2024). The unique properties of Big tau in the visual system. Cytoskeleton.

[19] Chung D.C., Deng X., Yalamanchili H.K., Revelli J.P., Han A.L., Tadros B., Richman R., Dias M., Naini F.A., Boeynaems S. (2024). The big tau splice isoform resists Alzheimer’s-related pathological changes. bioRxiv.

[20] Nagao S.I., Kumamoto T., Masuda T., Ueyama H., Toyoshima I., Tsuda T. (1999). Tau expression in denervated rat muscles. Muscle Nerve.

[21] Gu Y., Oyama F., Ihara Y. (1996). Tau is widely expressed in rat tissues. J. Neurochem..

[22] Caillet-Boudin M.L., Buee L., Sergeant N., Lefebvre B. (2015). Regulation of human MAPT gene expression. Mol. Neurodegener..

[23] Lim G.B. (2023). Aggregation of big tau disrupts microtubules and causes diastolic dysfunction. Nat. Rev. Cardiol..

[24] Choi C.S., Gwin M., Voth S., Kolb C., Zhou C., Nelson A.R., deWeever A., Koloteva A., Annamdevula N.S., Murphy J.M. (2022). Cytotoxic tau released from lung microvascular endothelial cells upon infection with Pseudomonas aeruginosa promotes neuronal tauopathy. J. Biol. Chem..

[25] Bowles K.R., Pugh D.A., Oja L.M., Jadow B.M., Farrell K., Whitney K., Sharma A., Cherry J.D., Raj T., Pereira A.C. (2022). Dysregulated coordination of MAPT exon 2 and exon 10 splicing underlies different tau pathologies in PSP and AD. Acta Neuropathol..

[26] Ruiz-Gabarre D., Carnero-Espejo A., Avila J., Garcia-Escudero V. (2022). What’s in a Gene? The Outstanding Diversity of *MAPT*. Cells.

[27] Gottschalk A.C., Hefti M.M. (2022). The evolution of microtubule associated proteins—A reference proteomic perspective. BMC Genom..

[28] Corsi A., Bombieri C., Valenti M.T., Romanelli M.G. (2022). Tau Isoforms: Gaining Insight into *MAPT* Alternative Splicing. Int. J. Mol. Sci..

[29] Andreadis A. (2005). Tau gene alternative splicing: Expression patterns, regulation and modulation of function in normal brain and neurodegenerative diseases. Biochim. Biophys. Acta.

[30] Fischer I. (2022). Evolutionary perspective of Big tau structure: 4a exon variants of MAPT. Front. Mol. Neurosci..

[31] Rouzier R., Rajan R., Wagner P., Hess K.R., Gold D.L., Stec J., Ayers M., Ross J.S., Zhang P., Buchholz T.A. (2005). Microtubule-associated protein tau: A marker of paclitaxel sensitivity in breast cancer. Proc. Natl. Acad. Sci. USA.

[32] Souter S., Lee G. (2010). Tubulin-independent tau in Alzheimer’s disease and cancer: Implications for disease pathogenesis and treatment. Curr. Alzheimer Res..

[33] Omar M.I., Roobol M.J., Ribal M.J., Abbott T., Agapow P.M., Araujo S., Asiimwe A., Auffray C., Balaur I., Beyer K. (2020). Introducing PIONEER: A project to harness big data in prostate cancer research. Nat. Rev. Urol..

[34] Benatar M., Ostrow L.W., Lewcock J.W., Bennett F., Shefner J., Bowser R., Larkin P., Bruijn L., Wuu J. (2024). Biomarker Qualification for Neurofilament Light Chain in Amyotrophic Lateral Sclerosis: Theory and Practice. Ann. Neurol..

[35] Fischer I., Baas P.W. (2020). Resurrecting the Mysteries of Big Tau. Trends Neurosci..

[36] Fischer I. (2023). Big Tau: What We Know, and We Need to Know. eNeuro.

[37] Fischer I., Baas P.W. (2025). Analyses of exon 4a structure reveal the properties of Big tau related to distribution, function and aggregation. Front. Mol. Neurosci..

[38] Barghorn S., Mandelkow E. (2002). Toward a unified scheme for the aggregation of tau into Alzheimer paired helical filaments. Biochemistry.

[39] Tomoo K., Yao T.M., Minoura K., Hiraoka S., Sumida M., Taniguchi T., Ishida T. (2005). Possible role of each repeat structure of the microtubule-binding domain of the tau protein in in vitro aggregation. J. Biochem..

[40] Jeganathan S., Hascher A., Chinnathambi S., Biernat J., Mandelkow E.M., Mandelkow E. (2008). Proline-directed pseudo-phosphorylation at AT8 and PHF1 epitopes induces a compaction of the paperclip folding of Tau and generates a pathological (MC-1) conformation. J. Biol. Chem..

[41] Friedhoff P., Schneider A., Mandelkow E.M., Mandelkow E. (1998). Rapid assembly of Alzheimer-like paired helical filaments from microtubule-associated protein tau monitored by fluorescence in solution. Biochemistry.

[42] Jeganathan S., von Bergen M., Brutlach H., Steinhoff H.J., Mandelkow E. (2006). Global hairpin folding of tau in solution. Biochemistry.

[43] Souter S., Lee G. (2009). Microtubule-associated protein tau in human prostate cancer cells: Isoforms, phosphorylation, and interactions. J. Cell. Biochem..

[44] Fischer I., Baas P. (2025). Analyses of exon 4a structure reveal unique properties of Big tau. bioRxiv.

[45] Zhang W., Tarutani A., Newell K.L., Murzin A.G., Matsubara T., Falcon B., Vidal R., Garringer H.J., Shi Y., Ikeuchi T. (2020). Novel tau filament fold in corticobasal degeneration. Nature.

[46] Mammeri N.E., Dregni A.J., Duan P., Hong M. (2024). Structures of AT8 and PHF1 phosphomimetic tau: Insights into the posttranslational modification code of tau aggregation. Proc. Natl. Acad. Sci. USA.

[47] Kirimtay K., Huang W., Sun X., Qiang L., Wang D.V., Sprouse C.T., Craig E.M., Baas P.W. (2025). Tau and MAP6 establish labile and stable domains on microtubules. iScience.

[48] Sun X., Yu W., Baas P.W., Toyooka K., Qiang L. (2024). Antagonistic roles of tau and MAP6 in regulating neuronal development. J. Cell Sci..

[49] Mason-Chalmers K., Sachdeva A., Kolk G., Touchon J.C., Donhauser Z.J. (2025). Tau Aggregation is Altered by Variations in its Projection Domain. bioRxiv.

[50] Wei M.L., Andreadis A. (1998). Splicing of a regulated exon reveals additional complexity in the axonal microtubule-associated protein tau. J. Neurochem..

[51] Lapointe N.E., Horowitz P.M., Guillozet-Bongaarts A.L., Silva A., Andreadis A., Binder L.I. (2009). Tau 6D and 6P isoforms inhibit polymerization of full-length tau in vitro. Biochemistry.

[52] Strang K.H., Golde T.E., Giasson B.I. (2019). MAPT mutations, tauopathy, and mechanisms of neurodegeneration. Lab. Investig..

