# Supplementary material for: Big Tau: Structure, Evolutionary Divergence, and Emerging Roles in Cytoskeletal Dynamics and Tauopathies
Source: Cells. 2026 Jan 27;15(3):241. doi: 10.3390/cells15030241 (PMC12896622; doi:10.3390/cells15030241)
Supplement: Supplementary file 1 [file cells-15-00241-s001.zip › cells-4098818-supplementary.pdf]

Supplemental data: Big Tau: Structure, Evolutionary Divergence, and Emerging Roles in Cytoskeletal Dynamics and Tauopathies

Table S1 Summary

Feature / Property	LMW tau	Big tau (with exon 4a)
**Size**	~45-60 kDa	95-110 kDa
**Localization**	CNS neurons (e.g., cortical, hippocampal)	PNS neurons, select CNS neurons (e.g., cerebellum)
**Microtubule Binding**	Stronger – repeats more accessible	Reduced – steric hindrance from exon 4a
**Axonal Transport**	Supports fast dynamics, plasticity	Suited for long, less plastic axons
**Aggregation Propensity**	High – exposed PHF motifs	Low – exon 4a shields PHF motifs
**Pathological Role**	Central in tauopathies	Rarely implicated in tau aggregates
**Evolutionary Trade-off**	High plasticity but aggregation risk in CNS	Lower tau aggregation risk but reduced adaptability
**Functional Advantage**	Rapid cytoskeletal remodeling, synaptic plasticity	Reduced axonal plasticity, reduced tau misfolding
**Vulnerability**	Susceptible to hyperphosphorylation and seeding	Protective against pathological aggregation

Legend Table S1

This is a graphical abstract of the review with a summary of the differences between Big tau and LMW tau.

Table S2: Exon-by-exon conservation table (human MAPT exons 1 → 13)

(Percent identity = amino-acid identity vs human)

**Exon**	**Functional Domain**	**Primate Gorilla**	**Rodent Mouse**	**Bird Chicken**	**Amphibian Xenopus**	**Fish Zebrafish**	**Agnathan Lamprey**
**1-4**	N-Terminal	98-99	75-90	45-60	30-40	20-30	15-25
**4a**	**Big Tau Specific**	**95-98**	**56**	**25-30**	**15-25**	**10-20**	**None**
**5-8**	Mid-Region/Projection	98-99	75-90	45-60	30-40	20-30	15-25
**9-12**	MTBD (R1-4)	99-100	97-99	85-95	80-90	70-85	60-70
**13**	C-Terminus	99-100	90-95	70-85	60-70	45-60	35-50

Legend Table S2
The table, which shows the approximate sequence identity of different vertebrate species relative to human tau exon by exons, illustrates the very low conservation of exon 4a and the high conservation of the MTBD. The approximate ranges are simplified presentations for the different vertebrate orders clustered into 5 general domains.
